# The Effect of Mindfulness on Self‐Management Behaviors in Inflammatory Bowel Disease Patients: The Suppression Effect of Ego Depletion

**DOI:** 10.1155/nrp/2043487

**Published:** 2026-05-12

**Authors:** Wen Zhang, Liyun Shang, Chun Gao, Su E. Yan, Xueqin Pang, Fanzhen Kong, Jie Gu

**Affiliations:** ^1^ Department of Gastroenterology, The First Affiliated Hospital of Soochow University, Suzhou, China, sdfyy.cn; ^2^ Department of Nursing, The First Affiliated Hospital of Soochow University, Suzhou, China, sdfyy.cn; ^3^ Department of Nursing, Suzhou Guangji Hospital, The First Affiliated Guangji Hospital of Soochow University, Suzhou, China

**Keywords:** IBD nursing, mindfulness, self-management behaviors, suppression effect

## Abstract

**Background:**

Based on ego depletion theory, this study investigates the mediating role of ego depletion in the relationship between mindfulness and self‐management behaviors in inflammatory bowel disease (IBD) patients, providing a reference for improving self‐management strategies.

**Methods:**

Using convenience sampling, IBD inpatients from the gastroenterology department of a tertiary hospital in Suzhou were selected as participants. Data were collected through the General Information Questionnaire, Mindful State Questionnaire (MSQ), Ego Depletion Scale, and IBD Self‐Management Behavior Questionnaire. Structural equation modeling (SEM) was applied to analyze the mediating effect of ego depletion between mindfulness and self‐management behaviors.

**Results:**

The model fit well (CFI = 0.97, RMSEA = 0.08). Mindfulness showed a direct positive relationship with self‐management (*β* = 0.45, *p* < 0.01) but was also associated with higher levels of ego depletion (*β* = 0.24, *p* < 0.01), which had a negative relationship with self‐management (*β* = −0.26, *p* < 0.01). A significant negative indirect effect was found (*β* = −0.065, 95% CI [−0.117, −0.027]), confirming ego depletion’s suppressor role.

**Conclusion:**

Ego depletion acts as a suppressor in the relationship between mindfulness and self‐management in this sample of hospitalized IBD patients. These findings suggest that mindfulness may be associated with increased feelings of ego depletion in this clinical population, which in turn undermines self‐management. This highlights a complex interplay where the beneficial direct association of mindfulness on behavior is partially offset by its relationship with resource depletion.

## 1. Introduction

Inflammatory bowel disease (IBD), encompassing two major subtypes Crohn’s disease and ulcerative colitis, is a chronic relapsing disorder characterized by persistent intestinal inflammation [1]. Globally, the incidence of IBD has been escalating annually, with China experiencing a particularly rapid surge in disease prevalence. It is projected that by 2025, China’s IBD patient population will reach 1.5 million, positioning the country among those with the highest disease burden worldwide [2, 3]. Patients chronically endure debilitating symptoms including abdominal pain, diarrhea, and fatigue, while simultaneously confronting psychological burdens such as anxiety and depression caused by disease recurrence. These compounded challenges lead to impaired social functioning and significant deterioration in quality of life [4], thereby posing formidable demands on patients’ self‐management capabilities.

Given the protracted disease course and absence of curative treatments for IBD, long‐term self‐management behaviors in patients—such as medication adherence, dietary regulation, and symptom monitoring—have emerged as critical determinants of disease prognosis [5]. Effective self‐management is associated with favorable health‐related outcomes, including reduce inflammation, alleviated symptom burden, improved psychological/physical well‐being, decreased relapse rates, and enhanced quality of life [5, 6]. However, existing studies reveal that only approximately 30%–50% of IBD patients consistently engage in effective self‐management practices, underscoring the imperative to systematically investigate the underlying influencing factors.

In recent years, the role of psychosocial factors in chronic disease management has garnered increasing attention. Mindfulness—a cognitive regulatory capacity centered on non‐judgmental awareness—has been demonstrated to promote health behaviors by mitigating stress and enhancing emotional regulation [7]. Among IBD patients, mindfulness levels show a positive correlation with disease adaptation capacity, yet the specific pathways through which mindfulness influences self‐management behaviors remain poorly elucidated.

A relevant theoretical framework is ego depletion theory, which posits that individuals possess limited self‐regulatory resources that can be depleted by prolonged cognitive or emotional effort [8]. Chronic disease management, with its ongoing demands for vigilance and adjustment, represents a potent source of such depletion. Traditionally, mindfulness is viewed as a resource‐conserving or replenishing practice. However, in the context of active IBD—where patients must maintain awareness amidst persistent discomfort and distress—the very act of mindful observation may itself be cognitively demanding. This suggests a more complex relationship: mindfulness could potentially coexist with, or even transiently heighten, feelings of ego depletion, especially when practiced without adequate support or in high‐stress periods [9]. This nuanced perspective remains under‐explored in IBD populations.

Current research exhibits three critical limitations: First, existing literature predominantly focuses on the direct effects of isolated variables, such as disease acceptance, social support, on self‐management behaviors, with scant attention to the dynamic depletion of psychological resources. Second, while mindfulness‐based interventions demonstrate potential in alleviating psychological distress among IBD patients, their long‐term efficacy and mechanistic pathways in enhancing self‐management behaviors require rigorous empirical validation. Third, current nursing intervention protocols overemphasize health education while neglecting the imperative of psychological resource restoration, thereby failing to address patients′ profound needs for sustainable behavioral change.

Building upon this theoretical framework, the present study proposes the following directional hypotheses based on existing theory:

 H1: Mindfulness is positively associated with self‐management behaviors in IBD patients.
 H2: Ego depletion is negatively associated with self‐management behaviors.

In addition, given the complex and potentially demanding nature of mindful self‐regulation in chronic illness, the relationship between mindfulness and ego depletion is addressed as a research question:

RQ1: What is the association between mindfulness and ego depletion in this population, and does ego depletion mediate the relationship between mindfulness and self‐management?

This investigation aims to unravel the pathways linking dynamic psychological resource depletion on behavioral outcomes, thereby providing a theoretical foundation for developing nursing interventions centered on “mindfulness training‐resource conservation” strategies.

## 2. Materials and Methods

### 2.1. Participants and Sample Size

Using convenience sampling, 237 eligible IBD inpatients from a tertiary hospital in Suzhou were recruited. Inclusion criteria: Age ≥ 18 years; Confirmed IBD diagnosis per the 2020 Chinese Society of Gastroenterology IBD diagnostic criteria; Fluent in Chinese and able to provide informed consent. Exclusion criteria: Comorbid intestinal diseases or malignancies; Severe cardiovascular, hepatic, renal, neurological, or psychiatric disorders; Employment in psychology or recent psychological interventions.

The sample size was calculated as 5–10 times the number of variables (26 variables). Accounting for a 10% attrition rate [10], the sample size was 143–286. A final sample of 237 participants was successfully recruited, meeting this target.

### 2.2. Measures

The General Information Questionnaire was self‐designed by the research team and comprised two components: (1) demographic data, including age, gender, marital status, educational level, employment/student status, and insurance coverage; (2) disease‐related data, encompassing IBD subtype, disease duration, history of IBD‐associated surgeries.

The Mindful State Questionnaire (MSQ) was employed to assess state mindfulness levels in IBD patients. Developed by Tanay and Bernstein [11]in 2013, this scale comprises 21 items rated on a 5‐point Likert scale, ranging from 1 (Never) to 5 (Always), with total scores spanning 21–105. Higher scores indicate stronger state mindfulness during specific tasks. The MSQ has demonstrated good reliability and validity, with a Cronbach’s *α* coefficient > 0.90. The scale demonstrated excellent reliability in this study (Cronbach’s *α* = 0.974).

The Ego Depletion Scale was revised by Lin et al. [12] based on prior self‐control scales developed by earlier scholars. This unidimensional 5‐item scale has been widely adopted in research to measure ego depletion. Representative items include “I feel mentally exhausted” and “I am unable to focus my attention now.” In this study, the same scale was utilized to assess ego depletion in participants. In this study, Cronbach’s *α* = 0.940.

The IBD Self‐Management Behavior Questionnaire was developed by Shang et al. [13]. This 36‐item instrument encompasses seven dimensions: medication management, dietary management, disease monitoring, emotional management, exercise management, daily life management, and resource utilization. Responses are rated on a 5‐point Likert scale, with total scores ranging from 36 to 180. Higher scores indicate better self‐management behaviors. The questionnaire demonstrated good reliability and validity, with a Cronbach’s *α* coefficient of 0.95 and a content validity index (CVI) of 0.93, confirming its effectiveness in evaluating self‐management behaviors in IBD patients. The scale showed excellent internal consistency here (Cronbach’s *α* = 0.970).

### 2.3. Ethics Committee Approval and Informed Consent

This study was approved by the Ethics Committee of The First Affiliated Hospital of Soochow University (Approval No. 2024‐Lun Shen Pi‐265. Date: July 15, 2024). All participants provided written informed consent prior to enrollment. No financial compensation was provided to participants. To ensure confidentiality, all questionnaires were collected anonymously, and data were stored in coded form on an encrypted, password‐protected computer accessible only to the research team.

### 2.4. Statistical Analysis

Data were analyzed using SPSS 27.0 and AMOS 24.0. Descriptive statistics (means, standard deviations) summarized the scores for mindfulness, ego depletion, and self‐management behaviors. Pearson correlation analysis examined bivariate relationships. The hypothesized mediation model was tested using structural equation modeling (SEM) with maximum likelihood estimation in AMOS. Model fit was assessed using *χ* ^2^/df, goodness‐of‐fit index (GFI), Tucker–Lewis index (TLI), comparative fit index (CFI), and root mean square error of approximation (RMSEA). The mediating role of ego depletion was tested using the bias‐corrected bootstrap method with 5000 resamples; a 95% confidence interval not containing zero indicated a significant effect. The significance level was set at *α* = 0.05.

## 3. Results

### 3.1. Participant Characteristics

A total of 249 questionnaires were distributed, with 237 valid responses collected (effective response rate: 95.18%). The sample comprised 70.5% males, 62.5% aged ≤ 40 years, and 67.1% married participants. Clinically, 80.2% had Crohn’s disease, 50.6% reported a disease duration 1–5 years, and 41.4% had undergone IBD‐related surgeries. Univariate analysis revealed no significant differences in self‐management behavior scores across demographic or disease‐related factors (*p* > 0.05). Detailed results are presented in Tables 1 and 2.

**TABLE 1:** Demographic and disease characteristics of participants (*n* = 237).

Variables	Variable classification	*n*	%
Age (year)	≤ 30	77	32.5
	31–40	71	30
	41–50	56	23.6
	> 50	33	13.9

Gender	Male	167	70.5
	Female	70	29.5

Educational level	Lower secondary education or less	34	14.3
	Academic/vocational	100	42.2
	Bachelor’s degree or higher	103	43.5

Marital status	Unmarried	72	30.4
	Married	159	67.1
	Divorced/widowed	6	2.5

Employment/student status	Continued academic/professional engagement	167	70.5
	Alternating phases of study/work and medical leave	39	16.5
	Sick leave for academic/professional obligations	31	13.1

Insurance coverage	Have insurance	235	99.2
	Self‐financing	2	0.8

Disease duration (year)	< 1	20	8.4
	1–5	120	50.6
	6–10	55	23.2
	> 10	42	17.7

History of IBD‐associated surgeries	No	139	58.6
	Yes	98	41.4

IBD subtype	Crohn’s disease	190	80.2
	Ulcerative colitis	47	19.8

Abbreviation: IBD, inflammatory bowel disease.

**TABLE 2:** Univariate analysis of self‐management behaviors (*n* = 237).

Variables	Variable classification	Mean	SD	*F*/*t*	*p* value
Educational level	Lower secondary education or less	128.56	35.409	0.035	0.966
	Academic/Vocational	127.5	29.511		
	Bachelor’s degree or higher	126.94	31.858		

Marital status	Unmarried	126.22	29.679	0.224	0.799
	Married	127.67	32.058		
	Divorced/widowed	134.83	34.126		

Employment/student status	Continued academic/professional engagement	127.91	30.125	0.183	0.833
	Alternating phases of study/work and medical leave	127.79	31.003		
	Sick leave for academic/professional obligations	124.23	38.144		

Disease duration (year)	< 1	129.25	41.422	0.347	0.791
	1–5	127.98	31.314		
	6–10	123.8	27.575		
	> 10	129.62	31.16		

Age (year)	≤ 30	128.35	31.205	0.377	0.769
	31–40	127.2	28.734		
	41–50	124.18	31.816		
	> 50	131.15	36.485		

Gender	Male	129.15	30.41	1.324	0.187
	Female	123.26	33.18		

Insurance coverage	Yes	127.71	31.26	1.589	0.113
	No	92.50	12.02		

History of IBD‐associated surgeries	No	126.12	31.50	−0.757	0.450
	Yes	129.24	31.08		

IBD subtype	Crohn’s disease	128.31	30.41	0.886	0.377
	Ulcerative colitis	123.79	34.79		

Abbreviations: IBD, inflammatory bowel disease; SD, standard deviation.

### 3.2. Descriptive Statistics and Correlations

As shown in Table 3, the total score of the Mindfulness State Questionnaire (MSQ) was 52.98 ± 21.96, and the Ego Depletion Scale total score was 10.10 ± 5.23. The IBD Self‐Management Behavior Questionnaire yielded an overall score of 127.41 ± 31.30, with subscale scores as follows: Medication Management (19.22 ± 5.08), Dietary Management (29.78 ± 9.14), Symptom Monitoring (15.96 ± 4.19), Emotional Management (21.84 ± 6.38), Exercise Management (10.18 ± 3.31), Daily Life Management (13.51 ± 4.17), and Resource Utilization (16.92 ± 5.09).

**TABLE 3:** Correlation matrix of mindfulness, ego depletion, and self‐management behaviors (*n* = 237).

Variable	Mean	Mindfulness	Ego depletion	Medication management	Dietary management	Symptom monitoring	Emotional management	Exercise management	Daily life management	Resource utilization	Total self‐management behavior
Mindfulness	52.98 ± 21.96	1									
Ego depletion	10.10 ± 5.23	0.247^∗∗^	1								
Medication management	19.22 ± 5.08	0.378^∗∗^	−0.086	1							
Dietary management	29.78 ± 9.14	0.247^∗∗^	−0.096	0.615^∗∗^	1						
Symptom monitoring	15.96 ± 4.19	0.329^∗∗^	0.032	0.641^∗∗^	0.595^∗∗^	1					
Emotional management	21.84 ± 6.38	0.380^∗∗^	−0.161^∗^	0.661^∗∗^	0.606^∗∗^	0.648^∗∗^	1				
Exercise management	10.18 ± 3.31	0.303^∗∗^	−0.091	0.573^∗∗^	0.657^∗∗^	0.554^∗∗^	0.706^∗∗^	1			
Daily life management	13.51 ± 4.17	0.224^∗∗^	−0.205^∗∗^	0.641^∗∗^	0.679^∗∗^	0.567^∗∗^	0.706^∗∗^	0.699^∗∗^	1		
Resource utilization	16.92 ± 5.09	0.345^∗∗^	−0.126	0.612^∗∗^	0.617^∗∗^	0.622^∗∗^	0.765^∗∗^	0.685^∗∗^	0.758^∗∗^	1	
Total self‐management behavior	127.41 ± 31.30	0.373^∗∗^	−0.128^∗^	0.808^∗∗^	0.855^∗∗^	0.779^∗∗^	0.868^∗∗^	0.813^∗∗^	0.852^∗∗^	0.855^∗∗^	1

^∗^Significant correlation at *α* = 0.05 (two‐tailed).

^∗∗^Significant correlation at *α* = 0.01 (two‐tailed).

### 3.3. Correlation Analysis of Mindfulness, Ego Depletion, and Self‐Management Behavior Scale Scores in Patients With IBD

Further Pearson correlation analysis revealed that mindfulness was significantly positively correlated with ego depletion, IBD self‐management behaviors, and scores across multiple subdomains (emotional management, exercise management, daily life management, and resource utilization). Conversely, ego depletion showed significant negative correlations with IBD self‐management behaviors and all its subdomains. Detailed results are presented in Table 3.

### 3.4. Path Analysis of the Impact of Mindfulness and Ego Depletion on Self‐Management Behaviors

The hypothesized mediation model was tested using the maximum likelihood estimation method in AMOS. The initial model fit indices suggested room for improvement: *χ* ^2^/df = 3.22, GFI = 0.93, TLI = 0.94, CFI = 0.95, and RMSEA = 0.10. Although some indices (e.g., GFI, CFI) were acceptable, the RMSEA value exceeded the 0.08 benchmark, indicating a suboptimal fit to the data.

To achieve a more parsimonious model, post hoc modifications were conducted guided by the modification indices (MIs). The modifications adhered to a principled approach: only one parameter was freed at a time, and any change required a plausible theoretical justification to avoid capitalizing on chance. The MIs suggested adding covariances between the error terms of two pairs of observed variables within the “self‐management behaviors” latent construct. Specifically, a high MI was found between the error terms for “medication management” and “symptom monitoring” (e3 and e5). This correlation is theoretically justifiable, as in chronic disease management, strict adherence to medication and vigilant monitoring of symptoms are often co‐occurring and intrinsically linked self‐management behaviors. Furthermore, a high MI was indicated between the error terms for “diet management” and “emotional management” (e4 and e6). This can be explained by the well‐established bidirectional relationship between dietary habits and emotional states, where dietary choices influence mood and vice versa, leading to correlated measurement errors.

Based on this theoretical rationale, covariances were sequentially added between e3 and e5, and between e4 and e6. The fit indices of the modified model demonstrated a significant improvement and all met the standard criteria for good model fit: *χ* ^2^/df = 2.63, RMSEA = 0.08, GFI = 0.95, TLI = 0.95, CFI = 0.97. Compared to the initial model, the modified model showed a notable decrease in RMSEA. Detailed fit indices are presented in Table 4. Critically, these modifications pertained only to the measurement model and did not alter the core structural paths, preserving the theoretical integrity of the hypothesized relationships. All subsequent analyses are based on this final model.

**TABLE 4:** Model fit indices for the structural equation model (*n* = 237).

Index	Reference standard	Initial model	Final model
*χ* ^2^/df	1–3 = Excellent, 3–5 = Good	3.22	2.63
RMSEA	< 0.05 = Excellent, < 0.08 = Good	0.10	0.08
GFI	> 0.9 = Excellent, > 0.8 = Good	0.93	0.95
TLI	> 0.9 = Excellent, > 0.8 = Good	0.94	0.95
CFI	> 0.9 = Excellent, > 0.8 = Good	0.95	0.97

*Note:* CMIN/DF = Chi‐square minimum/degrees of freedom.

Abbreviations: CFI, comparative fit index; GFI, goodness‐of‐fit index; RMSEA, root mean square error of approximation; TLI, Tucker–Lewis index.

The standardized path coefficients for the final structural model are presented in Table 5. All hypothesized paths were statistically significant (*p* < 0.01). Specifically, mindfulness was significantly and positively associated with ego depletion (*β* = 0.24). Concurrently, mindfulness had a direct positive association with self‐management behaviors (*β* = 0.45), while ego depletion significantly and negatively predicted self‐management behaviors (*β* = −0.26).

**TABLE 5:** Standardized path coefficients for the final structural model (*n* = 237).

Path relationship test	Standardized estimate	Unstandardized estimate	S.E.	C.R.	*p* value
Ego depletion <‐‐‐ Mindfulness	0.24	0.06	0.015	3.909	< 0.01
Self‐management behavior <‐‐‐ Mindfulness	0.45	0.08	0.012	6.639	< 0.01
Self‐management behavior <‐‐‐ Ego depletion	−0.26	−0.19	0.047	−4.105	< 0.01

**FIGURE 1:**
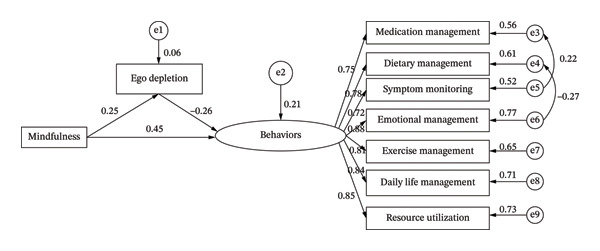
Paths depicting the relationships among mindfulness and ego depletion on self‐management behaviors in patients with inflammatory bowel disease.

The mediation analysis was conducted using the bias‐corrected bootstrap method. With 5000 bootstrap resamples, the 95% confidence intervals for both the direct effect (0.448, 95% CI: [0.332, 0.561]) and the indirect effect (−0.065, 95% CI: [−0.117, −0.027]) of mindfulness on self‐management behaviors did not include zero (Table 6). This confirms the statistical significance of the mediating pathway through ego depletion. Crucially, the negative value of the indirect effect indicates that ego depletion functions as a suppressor variable in this relationship (see Figure 1). The total effect was also significant (0.383, 95% CI: [0.253, 0.508]).

**TABLE 6:** Bootstrap mediation effect test results (*n* = 237).

Variable	Standardized estimate	Bootstrap S.E.	95% CI
Indirect effect	−0.065	0.023	[−0.117, −0.027]
Direct effect	0.448	0.059	[0.332, 0.561]
Total effect	0.383	0.014	[0.253, 0.508]

## 4. Discussion

This study examined the interrelationships among mindfulness, ego depletion, and self‐management behaviors in a sample of hospitalized IBD patients from a tertiary hospital in Suzhou and tested the mediating role of ego depletion. Consistent with hypothesis H1, mindfulness was positively correlated with self‐management behaviors, underscoring its potential as a facilitative factor in chronic disease management. Studies have demonstrated that mindfulness enhances individuals’ awareness of mind‐body states and fosters nonjudgmental acceptance, thereby improving their ability to cope with disease‐related stressors [14]. The direct link between mindfulness and self‐management is consistent with the cognitive‐behavioral integration model. Neuroimaging evidence [15] indicates that mindfulness training enhances regulatory control of the anterior cingulate cortex over the limbic system, thereby providing biological plausibility for the pathways suggested by this study. Similarly, research on type 2 diabetes patients revealed that blended mindfulness‐based stress reduction programs significantly improved self‐management behaviors [16]. Additionally, mindfulness has been shown to positively modify dietary behaviors in individuals with obesity [17], which is consistent with the findings of this study.

Also, as hypothesized H2, ego depletion was negatively correlated with self‐management behaviors. This result reinforced the core premise of ego depletion theory and was consistent with findings across other chronic conditions, where depleted self‐regulatory resources correspond to reduced adherence and self‐care. For instance, Chew et al. [18] demonstrated in heart failure patients that effective self‐regulation conserves psychological resources and enhances self‐care behaviors. Similarly, Ma et al. [19] synthesized evidence showing that individuals with ego depletion tend to reduce health‐related behaviors, such as adopting less healthy diets and decreasing exercise engagement. Furthermore, a survey of type 2 diabetes patients revealed a significant negative correlation between ego depletion and health‐promoting behaviors, where the more pronounced the ego depletion, the fewer health‐promoting behaviors were observed [20].

The most salient and unexpected finding emerged in addressing our research question (RQ1). Contrary to the common view that mindfulness conserves or restores psychological resources, mindfulness was positively associated with ego depletion in this clinical sample. This positive link, coupled with the negative relationship between ego depletion and self‐management, resulted in ego depletion acting as a suppressor variable. The suppressing effect—a specific type of indirect effect model—manifests when the direction of the indirect effect opposes that of the direct effect, resulting in obscured total effects [21].

This finding is consistent with the hypothesis that the negative association of ego depletion “offsets” or “masks” part of the positive relationship of mindfulness on self‐management. When we control for ego depletion in the model, the direct positive association between mindfulness and self‐management became more clearly visible. Furthermore, the suppressing effect of ego depletion elucidated a unique dilemma in IBD behavioral management: even with well‐established mindfulness capacities, patients may succumb to “intention‐action discordance” due to cognitive fatigue when psychological resources are persistently depleted. This finding challenged the linear assumption of “knowledge dictates behavior” prevalent in traditional health education paradigms, highlighting the gatekeeper role of psychological energy reserves in behavioral translation. This finding offers insights into the intrinsic associations through which mindfulness is related to IBD self‐management behaviors. Similar mediating pathways, in which mindfulness is associated with health behaviors via ego depletion, have been observed in chronic conditions such as diabetes [22], corroborating the present results.

This finding invited a clinically nuanced interpretation. In the high‐demand context of active IBD—where patients must continually monitor symptoms and adjust behaviors—the intentional practice of mindful awareness may itself be cognitively effortful. Maintaining a non‐judgmental focus on discomfort and internal states can require sustained self‐regulatory effort, which may transiently heighten feelings of resource depletion, especially during symptom flares or in earlier phases of practice. This aligned with the broader principle that all acts of self‐control, even those aimed at adaptation, draw upon a limited resource pool [9]. Thus, for some IBD patients, mindfulness may not simply alleviate depletion; it may coexist with or even temporarily be associated with increased depletion.

In summary, our model suggests a possible pathway through which mindfulness relates to self‐management in this sample of hospitalized IBD patients: a direct positive pathway was partially counterbalanced by an indirect pathway via increased ego depletion. These findings are consistent with the need to consider psychological resource dynamics in chronic illness care and to develop interventions that support not only mindful awareness but also resource conservation and recovery.

## 5. Limitations and Future Research

However, this study has several limitations. First, the cross‐sectional design precludes causal inferences, longitudinal or experimental studies are needed for validation. Second, this study employed convenience sampling to recruit IBD inpatients from a tertiary hospital in Suzhou. The sample’s representativeness is limited in terms of geography, healthcare institution type, and patient type, which may restrict the generalizability of the findings to other regions, primary care settings, outpatients, or IBD populations from different cultural backgrounds. Therefore, the findings should be interpreted as specific to this hospitalized sample, and future research should adopt multi‐center, stratified sampling designs to enhance sample representativeness and the generalizability of results. Third, the limited sample size may affect statistical power, and future research should expand recruitment and incorporate moderating variables. Fourth, the Ego Depletion Scale used in this study contains only five items. Although it showed good reliability in this study, its breadth of content may be limited and may not fully encompass all aspects of the complex psychological construct of ego depletion across behavioral, cognitive, and emotional dimensions. Future research could employ more comprehensive, multi‐dimensional measurement tools, for instance, the State Self‐Control Capacity Scale, to assess ego depletion more precisely. Fifth, although the fit indices of the structural equation model in this study reached acceptable standards after modification (RMSEA = 0.08), the RMSEA value is at the upper limit of the “acceptable” range, indicating that the model fit to the data still has room for improvement. This may be related to sample size, measurement error, or the complexity of the model itself. Future research is needed to validate and refine this theoretical model with larger samples. Sixth, integrating physiological indicators, such as inflammatory biomarkers including C‐reactive protein (CRP) or interleukin‐6 (IL‐6), could further elucidate the biological mechanisms underlying mindfulness’ associations with IBD.

## Author Contributions

Conceptualization: Wen Zhang, Jie Gu, and Fanzhen Kong; data curation: Wen Zhang, Chun Gao, Su E Yan, and Liyun Shang; formal analysis: Wen Zhang; methodology: Wen Zhang; writing–original draft: Wen Zhang; resources: Jie Gu, Fanzhen Kong and Xueqin Pang; project administration: Jie Gu and Fanzhen Kong; supervision: Jie Gu and Fanzhen Kong; writing–review and editing: Jie Gu and Fanzhen Kong.

## Funding

No funding was received for this manuscript.

## Consent

The authors have nothing to report.

## Conflicts of Interest

The authors declare no conflicts of interest.

## Data Availability

The data presented in this study are available upon request from the corresponding author.
